# 肿瘤肺泡腔内播散与肺腺癌的临床病理特征及对肺癌外科治疗和预后影响的研究进展

**DOI:** 10.3779/j.issn.1009-3419.2019.06.06

**Published:** 2019-06-20

**Authors:** 贵东 屈, 云飞 施

**Affiliations:** 650032 昆明，昆明医科大学第一附属医院 Elderly Thoracic Surgery, the First Affiliated Hospital of Kunming Medical University, Kunming 650032, China

**Keywords:** 肿瘤肺泡腔内播散, 病理, 手术, 冰冻切片, 预后, Tumor spread through air spaces, Pathology, Surgery, Frozen-section, Prognosis

## Abstract

肿瘤肺泡腔内播散（tumor spread through air spaces, STAS）作为一种新的病理侵袭方式，与诸多临床病理因素紧密相关。在肺腺癌中，微乳头和实体型病理亚型与之关系最为密切；针对早期肺腺癌存在STAS，手术类型的治疗上肺叶切除似乎优于亚肺叶切除而获益，可能上调早期肺癌的病理分期；同时，STAS与鳞癌等非小细胞肺癌（non-small cell lung cancer, NSCLC）关系密切。此外，STAS的术中冰冻切片病理检测困难，亦有争议存在。STAS作为肿瘤复发的独立危险因素，亦是提示预后不良的重要因素，本文将STAS的研究现状和进展作一综述。

## Abstract (en)

Tumor spread through air spaces (STAS) as a new pathological invasion mode is closely related to many clinicopathological factors. In lung adenocarcinoma, micropapillary and solid pathological subtypes are most related; STAS for early stage lung adenocarcinoma, surgical type of lobectomy seems to benefit better than sublobar resection, which may up-regulate the pathological stage of early lung cancer; Moreover, STAS is closely related to squamous cell carcinoma and other non-small cell lung cancer (NSCLC). In addition, intraoperative frozen-section pathological detection of STAS is difficult and controversial. STAS as an independent risk factor for tumor recurrence is also an important factor indicating poor prognosis. This paper reviews the research status and progress of STAS.

肺癌是目前最常见的恶性肿瘤之一，肺癌的总体生存率不高，这与患者就诊时往往已发生转移而处于肿瘤晚期相关，其预后与肿瘤的分期紧密关联。近年来，随着高分辨计算机断层扫描（computed tomography, CT）对高危人群的筛查，早期非小细胞肺癌（non-small cell lung cancer, NSCLC）结节的检出率增加，更多人群获益，早期肺癌首选外科手术^[1]^，可明显提高肺癌的治愈率及生存率，但部分Ⅰ期NSCLC患者行肺癌根治术后仍有较高的复发和转移，预后差。随着病理研究的深入，在既往常见的淋巴转移、血行转移及局部种植转移之外，发现了被认为是另类浸润或转移方式-肿瘤肺泡腔内播散，STAS增加了早期肺癌转移和复发风险，是提示预后不良的重要因素。针对存在STAS的早期NSCLC行肺叶切除预后是否优于亚肺叶切除、肺癌T分期是否会受到影响、Ia期术后是否需要辅助化疗等问题值得探讨；其概念出现较晚，受关注度不高，尚未形成对指导肺癌治疗的临床意义，结合相关文献研究，现对STAS的发生率、临床及病理、分子特征、对早期肺腺癌预后的影响等研究进展作一综述。

## 1 STAS的定义及相关概念

STAS是由微乳头状簇、实性巢或肿瘤边缘以外的单个肿瘤细胞组成，进入周围肺实质的肺泡腔隙，并在2015年世界卫生组织肺癌病理分类中明确指出了STAS是一种新的肿瘤侵袭（转移）模式^[2]^；可理解为肿瘤细胞离开肺癌肿块边缘，进入到周边肺实质肺泡和毛细支气管内。在此之前，Onozato等^[3]^研究发现此现象，一个孤立的、大量的肿瘤细胞集合，呈模糊的微乳头状结构，存在于肺泡间隙，称之为肿瘤岛，肿瘤岛位于病变周围，与主要肿瘤之间至少隔有几个肺泡，见于肺腺癌。Kadota等^[4]^又对STAS的成分做了具体描述：单细胞被定义为肺泡腔内无粘性的单个肿瘤细胞；微乳头状簇被定义为在肺泡腔内没有中央纤维血管核心的乳头状结构；实性巢被定义为充满肺泡腔内的肿瘤细胞的实性集合；从形态学上看，这些肿瘤细胞位于肺泡间隙，如微乳头状簇、实性巢或与肺泡壁分离的单个肿瘤细胞，这与肿瘤细胞沿肺泡壁表面呈线性生长的lepidic（钉突样）生长不同；肿瘤细胞对肺泡腔的填充程度不同，从大量的细胞浸润到非常不明显的单个细胞或微乳头状簇，有时很难与肺泡巨噬细胞进行区分。国际肺癌研究学会（International Association for Study of Lung Cancer, IASLC）、美国胸科学会（American Thoracic Society, ATS）和欧洲呼吸学会（European Respiratory Society, ERS）公布了2011年肺腺癌的国际多学科新分类，并引入了“5%递增”的半定量概念，使得肺腺癌的综合治疗更加精准化^[5]^；Uruga等^[6]^借此对STAS进行了半定量评估，分为无STAS、低STAS（1个-4个单肿瘤细胞或STAS簇）、高STAS（≥5个单肿瘤细胞或STAS簇），这是首次对STAS进行标准化、定量评价，定量测量有助于组织学分类和风险分层，对指导后续研究意义重大。目前其名称并未统一，国内使用的名称为肺泡腔内播散、气腔内播散等。

## 2 STAS与肺腺癌的临床病理、分子及影像学特征的关系

尽管部分研究认为STAS易发生在吸烟^[3]^、和或男性吸烟^[7, 8]^人群中，但更多研究显示性别、吸烟及年龄均与STAS无关^[4, 6, 9-13]^。STAS与肺腺癌中微乳头或实体型显著相关^[3, 4, 6, 8, 12]^，还发现伴有实性或微乳头结构混合型腺癌中多出现STAS，很少出现于附壁型腺癌中^[13]^。STAS与胸膜浸润、淋巴血管浸润、肿瘤性坏死之间关系密切，淋巴管及血管浸润与之有显著相关性^[6, 12]^，然而部分研究却显示STAS与胸膜浸润无关^[4, 13]^；STAS还与晚期肿瘤分期、低组织学分化程度有关^[14]^。部分研究认为肿瘤直径可影响STAS发生率，在Ⅰ期肺腺癌患者中，Dai等^[15]^认为STAS发生率与肿瘤大小无关，日本学者Toyokawa等^[10]^认为肿瘤 > 2 cm、徐等^[13]^认为肿瘤≥2.7 cm时，出现STAS比例高；当肿瘤直径≤2 cm时，Kadota等^[4]^认为STAS阳性者与肿瘤大小无关，而Uruga等^[6]^认为高STAS与肿瘤≥1 cm有显著相关性；在Ⅰ期-Ⅳ期时，孙等^[12]^认为SATS与肿瘤≥3 cm相关。癌胚抗原水平亦可影响STAS发生率，有研究显示STAS与癌胚抗原高水平时有关^[7]^，当CEA > 3.2 ng/mL时与STAS关系密切^[10]^；而反对者研究认为STAS与癌胚抗原水平无关^[15]^。部分研究还显示了STAS与肿瘤突变状态的关系，患有STAS时多存在EGFR野生型，并可能出现*KRAS*突变、*BRAF*突变、*ALK*和*ROS1*基因重排几率增加^[3, 7, 8, 12]^。

手术切除前通过影像学可部分预测STAS特征，Toyokawa等^[10]^指出术前实变/肿瘤（C/T）比值≥0.25和最大标准摄取值（SUV_max_）≥2.5是STAS的独立预测指标；随后Toyokawa等^[9]^在术前CT影像学评估STAS做了尝试，单因素分析中，肿瘤直径、血管征、胸膜牵拉、毛刺征、分叶征分别与STAS关系密切，磨玻璃样结节（ground glass opacity, GGO）与之无相关性；多变量因素分析中，与有GGO成分而无切迹征相比，无GGO成分却存在切迹征的腺癌比值比（odds ratio, OR）高达5.01（95%CI:2.73-9.52, *P* < 0.01），说明了有切迹征却无GGO成分与STAS显著相关；在有实性成分时，STAS的出现频率高于不含STAS的腺癌。Shiono等^[7]^的研究则直接指出术前表现中，通过STAS与CT上的实性结节显著相关。

目前不同文献研究对STAS与肺腺癌的临床病理等特征之间作出了积极评价，多显示STAS与年龄、性别及吸烟无关，一致认为STAS多出现在微乳头或实体型肺腺癌中，然而其评价内容及标准不同的缘故，可能造成在病理分期、肿瘤浸润程度、肿瘤直径、分子特征等方面预测STAS具有差异化，同时CT影像亦预测STAS与实性成分相关，然而目前并没有良好预测STAS的特异性指标。

## 3 STAS在早期肺腺癌手术中的预后意义

早期肺腺癌伴有STAS时预后不良，存在STAS的早期肺腺癌患者预后亦会受到手术类型的影响。Onozato等^[3]^研究显示肿瘤岛组和无肿瘤岛组5年无复发生存率（recurrence-free survival, RFS）分别为44.6%和74.4%，即使在Ia期，有肿瘤岛的患者超过一半在5年内复发；此外含有5%以上微乳头型是肺腺癌患者亚肺叶切除术后复发的重要预测因素，但并没有说明其与STAS的组织学关系。Kadota等^[4]^的研究认为STAS阳性者5年复发累积发生率（cumulative incidence of recurrence, CIR）为42.6%，明显高于STAS阴性5年CIR10.9%，接受亚肺叶切除时患者的总生存期（overall survival, OS）与STAS的相关性有统计学意义（P=0.045），而肺叶切除组中STAS阳性患者与STAS阴性患者的5年OS无差异；存在STAS是Ⅰ期肺腺癌经有限切除治疗后复发的重要危险因素，该研究还发现含有微乳头成分的肿瘤患者行肺叶切除术相比有限切除明显获益；总之，STAS是复发的独立危险因素。Uruga等^[6]^发现在行肺叶切除的患者中，较高的STAS与较短的RFS相关；在亚肺叶切除中，STAS与RFS之间无统计学意义；增加STAS与缩短OS有显著相关性。在Toyokawa等^[10]^的定量评价中，术后随访5年，亚肺叶切除组STAS与OS明显相关，而行肺癌根治术组STAS与OS无明显差异；亚肺叶切除与肺癌根治术组中，STAS对RFS均无明显影响；然而该研究认为针对STAS的早期肺癌行肺叶切除能否为患者带来更多获益，其意义尚不明确。其后Toyokawa等^[9]^又进一步研究STAS在CT中的表现时发现STAS阳性肺腺癌患者的RFS和OS短于STAS阴性组；经手术切除的STAS阳性腺癌患者的RFS和OS短于无STAS时，特别是有限切除STAS阳性腺癌患者术后生存率明显低于STAS阴性组，并推测STAS阳性腺癌为病理侵袭性癌。上述研究显示存在STAS的肺腺癌预后受到手术类型的影响，同时亦表明STAS是有限切除而非肺叶切除术患者复发的重要危险因素。然而Dai等^[15]^分析他们组并综合了其他人的数据，认为STAS与不良的生存结果密切相关，但却与手术类型无关。目前对早期肺腺癌在外科领域关注度最高的是肺叶切除或是亚肺叶切除（解剖性肺段切除和楔形切除），早期推荐的首选是行肺叶切除+纵隔淋巴结清扫或取样，目前越来越多的证据支持早期肺腺癌（≤2 cm）行亚肺叶切除+纵隔淋巴结取样其预后并不劣于肺叶切除术^[16, 17]^，并同时保留肺功能，特别是老年患者；但针对含有实性成分的肺癌具有侵袭性，肺叶切除加淋巴结清扫仍然是治疗实体成分、临床Ia期的肺腺癌的标准手术方法^[18]^，鉴于STAS与肺腺癌中含有微乳头或实体型成分时的密切关联，含有STAS的肺腺癌行肺叶切除加淋巴结清扫可能是最佳治疗手段。

Uruga等^[6]^同时发现在切除的小腺癌中有1/3的STAS高，STAS越高，RFS越差，大约20%的高STAS患者出现复发；STAS的数量可能是影响预后的重要因素，然而STAS在肿瘤中的数量并不是唯一对预后有影响的指标^[19]^，Kadota等还发现最远的STAS距肿瘤边缘的距离为1.7 cm，Dai等^[15]^也指出STAS与肿瘤边缘的最大估计距离为1.35 cm，故它与主要肿瘤肿块的距离亦需要受到关注，Warth等^[8]^评估STAS是否位于远离主要肿瘤肿块的 < 3个肺泡距离（“局限性”STAS）和位于远离主要肿瘤肿块 > 3个肺泡距离（“广泛”STAS）的预后，发现局限性和广泛性STAS患者的预后明显比无STAS患者差，但有局限性和广泛性STAS患者的预后没有差异；然而广泛的STAS与微乳头状占优势的肿瘤密切相关。此外，Lee等^[11]^认为STAS可能是肿瘤侵袭性的一个参数，STAS与胸外及胸内包括肺内部位复发密切相关；有限切除时STAS切缘不足可能导致局部复发，为实现STAS阳性肺癌的根治性切除，切缘应更宽^[14]^；然而当切缘距离 < 1.0 cm与局部复发风险密切相关，而切缘距离 > 2.0 cm的肿瘤则无局部复发，而不考虑其他因素，包括肿瘤STAS^[20]^。而从以往经验得知，外科医师在对早期肺癌行楔形切除时，往往会注意将切缘距离肿瘤至少2 cm，可降低复发风险，但对于STAS阳性的肺腺癌，切缘距离肿瘤可能远远超过2 cm；当然需要更多的数据，以确认STAS对临床治疗决策的潜在重大影响^[21]^。

Dai等^[15]^研究认为STAS阳性的肺腺癌（≤3 cm）患者的RFS和OS均低于无STAS的患者，将存在STAS的肺腺癌（≤3 cm）与Ib肺腺癌分组对比，RFS（*P*=0.091）和OS（*P*=0.443），可看出统计学无差异；在肺腺癌（≤2 cm）患者中，STAS对预后的影响很小，但在肺腺癌 > 2 cm-3 cm时可明显预后不佳；并认为肺腺癌（> 2 cm-3 cm）STAS阳性患者的RFS和OS与肺腺癌IB期患者相似，建议STAS可以作为分期系统中的一个因素来更准确地预测预后，特别是在ADC > 2 cm-3 cm时，并据此认为腺癌（> 2 cm-3 cm）STAS阳性患者可从术后化疗中获益。另外在Morimoto等^[22]^研究中发现一个有趣的现象，STAS阴性腺癌的无复发存活曲线与含有微乳头型的pT2腺癌的无复发存活曲线相当，而STAS阳性腺癌的无复发生存曲线与含有微乳头型的pT3型腺癌的无复发存活曲线相当。通过对Ia期肺腺癌中STAS的存在或缺失进行系统的评估，可以获得更精确的病理分期系统^[23]^。STAS可能被认为是肺癌分期的一个参数，尤其是对于肺腺癌^[24]^。

存在STAS的早期肺腺癌患者RFS和OS均较无STAS时低，在接受亚肺叶切除治疗的患者预后明显受到STAS的影响；STAS的数量越多可增加预后不良，STAS游离肿瘤边缘的距离远近可能不会影响预后，那么切缘距离超过2 cm就可使存在STAS的肺癌患者获益显然是有问题的，今后在对待含有STAS的早期肺腺癌外科处理方式上，肺叶切除术和亚肺叶切除、亚肺叶切除中不同切缘距离的预后分析仍需进一步研究；此外，STAS阳性可能是提升T分期的重要因素，应予以重视。

## 4 STAS与其他肺癌的关系

在肺鳞癌中，STAS病理形态均表现为实性巢，淋巴、血管浸润与STAS关系密切^[25, 26]^。Lu等^[25]^还得出STAS发生率随分期增加而增加；STAS与肿瘤的增殖有关，尤其Ki-67≥20%时；有STAS的患者远处和局部复发率及肺癌特异性死亡的发生率明显高于无STAS的患者，在OS上无明显差异，故STAS是复发和肿瘤特异性死亡的独立预测因子，而不是OS的独立预测因子。Yanagawa等^[26]^发现STAS在Ⅰ期、Ⅱ期、Ⅲ期中的发生率无明显差异；STAS仅有增加Ki-67高表达的趋势，p53亦与STAS状态无关；亚肺叶切除组患者的RFS和OS均明显低于无STAS组，肺叶切除术组STAS患者的RFS和OS均有下降趋势，但无统计学意义（*P*=0.056和*P*=0.111）；STAS仅仅是预测Ⅰ期肺鳞癌复发和预后的重要指标，在Ⅱ期、Ⅲ期时不起作用，而淋巴血管浸润和组织病理学亚型则是预测Ⅱ期、Ⅲ期复发的重要因素。Yokoyama等^[27]^对35例肺多形性癌（肉瘤样癌）的研究中，约占NSCLC的0.4%，STAS与梭形细胞癌、巨细胞癌、腺癌、鳞癌的病理特征无明显相关性，STAS与腺癌病理亚型之间也没有明显的关联，存在STAS的患者RFS和OS低于无STAS的患者，STAS是复发和总生存率低的独立危险因素，其样本量太小，证据尚显不足。可见STAS并不是肺腺癌独有的病理现象，同样存在于其他NSCLC中；STAS并不是一个罕见的发现，在决定正确的治疗策略时，特别是在外科手术中^[28]^，必须考虑到这一点；可是对于中央型鳞癌来说，与周围组织关系更为复杂，STAS存在与否往往不会影响手术类型，更应关注到术后辅助治疗能否获益。

STAS同样出现在肺鳞癌和肺多形性癌等中，其病理形态单一，临床病理特征评价亦存在差异化，但同样显示预后不良；如果STAS作为监测Ⅰ期肺鳞癌患者复发的重要指标，对于肺鳞癌患者多需行肺叶切除而不是亚肺叶切除治疗，那么其监测效果可能会受到手术类型的影响，故将STAS作为监测复发的指标应该还需要特定的标准。

## 5 STAS是否存在的争议与检测难点

目前有争议的是STAS是否存在和在所有情况下都是体内效应或可能是用刀切割肿瘤所引起的伪影。病理检测确实观察到STAS，现有数据表明STAS的发生率14.8%-61.8%不等，但是从不同的研究可得知，如Shiono等^[7]^的研究与孙平丽等^[12]^的研究STAS的发生率（14.8%:61.8%）可相差4倍，怎样去解释这一现象？Blaauwgeers等^[29]^研究认为是切割造成了STAS在肺泡腔内的扩散，漂浮的肿瘤细胞团簇在肺标本切片中的传播，称为“通过刀表面传播”，其代表了体外伪影，93%的松散组织碎片可以通过与组织处理相关的机械力来解释，以此认为2015年WHO对STAS的定义与解读是存在争议的；亦有人担心手术过程中的外科处理或吻合器技术可能会影响STAS的形成，这种现象被称为“通过外科手术传播”^[30]^。如果STAS只是手术切除后在体内诱发的伪影，那么我们如何解释STAS与预后和复发的显著关系？或许归于部分研究认为STAS与淋巴血管浸润关系密切；然而在Toyokawa等的研究中却发现亚肺叶切除组的STAS发生率（*n*=86, 37.2%）明显低于至少为肺叶切除组（*n*=241, 65.9%）；目前已有的研究表明，STAS是一种独立于淋巴血管侵袭的重要预后指标^[4, 26, 31]^。Warth等^[30]^观察到肺泡内巨噬细胞作为一个细胞群体存活的现象，同理推测肺泡壁的液体或瘤周水肿或出血可能会为STAS提供潜在能量供应，同时肺泡腔内的氧气或多或少是可用的；细胞培养实验亦表明，肺癌细胞可在细胞培养基中存活12 h以上，从而为STAS存在肺泡腔内提供依据。

STAS隐匿存在，切割亦可能造成播散，对其检测较困难，故目前并没有受到病理科及外科医师的足够重视。术中冰冻切片与石蜡切片的一致率为69.7%，腺泡和实体型最有可能被正确地诊断为主要的生长类型，只有11%-55%的病例能发现微乳头型；如果Ⅰ期肺腺癌的手术治疗将以其组织学亚型为指导，则病理医师在术中对冰冻切片取样的程度有潜在的影响^[32]^。冰冻切片标本中欠缺检测出STAS的能力，目前只有一项研究报告冰冻切片中STAS模式诊断的敏感性、特异性和准确性分别为71%、92.4%和80%^[33]^；最近关于冰冻切片组织学模式分类的准确性的证据表明，冰冻中识别一种微乳头生长模式具有很高的特异性，但敏感性很低，认为STAS可能会出现类似的情况^[31]^。由此可见对于STAS的病理检测，尤其是术中冰冻还需要更多的实践。

## 6 总结

目前，针对STAS的病理等因素和预后价值已经得到非常有意义的研究，可对临床治疗决策产生更积极的影响，建议STAS阳性患者行肺叶切除，而这需要术中病理医师快速准确评估，可以预见这将是一项艰巨的任务。目前依然有许多问题需要解决，STAS的病理评价标准并未统一，STAS能否上调病理分期并写入病理报告证据仍待完善，STAS阳性的患者亚肺叶切除后化疗能否获益还鲜有报道。总之，STAS作为一种新的病理侵袭方式，可用来评估早期肺癌的预后并适时干预临床治疗，当然还要做很多工作。
